# Energy expenditure responses to exercise training in older women

**DOI:** 10.14814/phy2.13360

**Published:** 2017-08-03

**Authors:** Xuewen Wang, Kimberly P. Bowyer, Ryan R. Porter, Charity B. Breneman, Sabra S. Custer

**Affiliations:** ^1^ Department of Exercise Science University of South Carolina Columbia South Carolina; ^2^ College of Nursing University of South Carolina Columbia South Carolina

**Keywords:** Energy compensation, energy expenditure, nonexercise activity thermogenesis, nonexercise physical activity, older

## Abstract

Previous studies have shown inconsistent findings regarding how structured exercise affects energy expenditure (EE). This study was designed to determine the changes in EE and physical activity following exercise training in older women. Nonobese (body mass index = 25.8 ± 3.4 kg·m^−2^) women (60–75 years, *n* = 72) completed a 4‐month supervised aerobic exercise training of lower‐ or higher‐dose (33.6 and 58.8 kJ·kg^−1^ body weight weekly, respectively) at 50–55% of heart rate reserve. Total daily EE (TDEE) by the doubly labeled water method, resting metabolic rate (RMR) via indirect calorimetry, and physical activity by accelerometer were determined before and at the end of exercise training. Nonexercise activity thermogenesis (NEAT) was calculated. Following exercise training, the changes in components of TDEE and total physical activity did not differ by group. In the entire sample, TDEE, RMR, NEAT and total physical activity did not change (*P* > 0.05 for all). However, a significant baseline physical activity × time interaction was found for several of the variables. Data were therefore stratified into tertiles of baseline physical activity. In the high tertile, TDEE remained unchanged, but total physical activity decreased (*P* = 0.012). In contrast, in the middle and low tertiles, NEAT remained unchanged, and total physical activity increased (*P* < 0.05 for both). In conclusion, aerobic exercise training did not change TDEE, RMR, NEAT, or total physical activity in this sample of older women. Exercise dose did not, but baseline physical activity levels might, influence EE responses and total physical activity changes.

## Introduction

Many conditions are related to an individual's ability to maintain energy balance. Energy balance involves dynamic processes, and components of total daily energy expenditure (TDEE) change when either side of the energy balance equation is perturbed. It has been shown previously that both resting and nonresting energy expenditure (EE) change in response to either caloric restriction or overfeeding (Leibel et al. 1995; Roberts et al. 1996a,b; Levine et al. 1999). The effect of structured exercise training on EE, however, is less clear. The traditional view is that TDEE increases with exercise training. However, this has been challenged in that a few exercise training studies reported less weight loss than predicted given the energy cost of exercise participation (Ross et al. 2000; Donnelly et al. 2003), which suggests a compensatory change in EE and/or energy intake. Further, the exercise training studies that have examined components of TDEE have reported inconsistent findings regarding whether exercise causes changes in TDEE or its components. Whether a compensatory reduction in non‐exercise activity thermogenesis (NEAT), a component of TDEE, occurs as a result of aerobic exercise training is highly debated in the literature (Washburn et al. 2014; Melanson 2017). NEAT is defined as the EE associated with all activities of daily living, such as fidgeting, maintaining posture, and ambulation, other than purposeful exercise (Levine and Kotz 2005; Melanson et al. 2013). Most studies in young to middle‐aged adults did not observe reductions in NEAT (McLaughlin et al. 2006; Hollowell et al. 2009; Turner et al. 2010; Rangan et al. 2011; Willis et al. 2014) or nonexercise physical activity (Meijer et al. 1991; Van Etten et al. 1997; Lynch et al. 2009; Rosenkilde et al. 2012; Willis et al. 2014). However, the findings in older adults (mean age >57 years) are mixed. Some studies have reported increased or unchanged nonexercise physical activity (Washburn and Ficker 1999; Fujita et al. 2003; Church et al. 2009), but others have reported decreased NEAT (Goran and Poehlman 1992; Morio et al. 1998) or nonexercise physical activity (Meijer et al. 1999, 2000) in response to aerobic exercise training.

Previous studies have used various methods to determine whether a compensatory response to exercise training occurred. Some studies used the doubly labeled water method (Meijer et al. 1991; Goran and Poehlman 1992; Van Etten et al. 1997; Colley et al. 2010; Willis et al. 2014) or whole‐room calorimeter (Morio et al. 1998) to determine TDEE, and subsequently calculated NEAT. Some studies used accelerometers to measure physical activity or estimate physical activity EE (Meijer et al. 1991, 1999, 2000; Van Etten et al. 1997; Washburn and Ficker 1999; Hollowell et al. 2009; Turner et al. 2010; Rangan et al. 2011; Di Blasio et al. 2012; Rosenkilde et al. 2012; Willis et al. 2014). Pedometer‐assessed steps (Church et al. 2009; Lynch et al. 2009), heart rate‐based calculations (Keytel et al. 2001; McLaughlin et al. 2006), and activity diaries (Morio et al. 1998; Fujita et al. 2003) also have been used in studies. Nonexercise physical activity and NEAT have been used interchangeably to interpret data. However, these two terms are different because wearable devices and activity diaries generally assess behavioral aspects of physical activity, and various physical activities are associated with differing amounts of energy cost. However, few studies have examined both NEAT and nonexercise physical activity simultaneously. Thus, it is difficult to draw a general conclusion based on studies that have used different methods and assessed different aspects of physical activity.

Further, most published studies in older adults used small sample sizes (≤20 per group) and/or were not randomized trials as summarized in a review (Washburn et al. 2014). However, it is important to know how NEAT and other TDEE components change in response to exercise training, given the epidemic of obesity and the fact that exercise is a common strategy for treating and preventing obesity. In this study, nonobese older women (60–75 years) were randomized to aerobic exercise training of 4 months at two different doses. The purpose of the study was to determine changes in components of TDEE following the exercise training, and to investigate whether any changes occurred in behavioral or physiological aspects of physical activity by simultaneously measuring EE and physical activity. Nonobese rather than obese participants were studied because we wanted to first examine EE responses under normal body weight regulation.

## Methods

### Design overview

The Women's Energy Expenditure in Walking Programs (WEWALK) study was a randomized trial designed to examine EE responses to 4 months of moderate‐intensity walking programs in older women. The study was registered at ClinicalTrials.gov (NCT01722136). The research protocol was approved by the University of South Carolina Institutional Review Board, and all participants provided written informed consent.

### Participants

Women from Columbia, SC and surrounding areas were recruited through local advertisement. Women were enrolled based on the following criteria: age (60–75 years), body mass index (18–30 kg·m^−2^), self‐reported stable weight (±3%) for the past 3 months, physically inactive (less than 20 min × 3 times per week of structured exercise) for the past 3 months, nonsmoking for the past year, and able to walk on a treadmill. The exclusionary criteria included: (1) self‐reported serious cardiovascular, metabolic, or respiratory diseases, or other conditions that might affect adhering to the protocol, affect exercise safety, or be aggravated by exercise, or, (2) medications known to affect exercise performance or metabolism, and (3) excess caffeine use (>500 mg·day^−1^).

**Figure 1:**
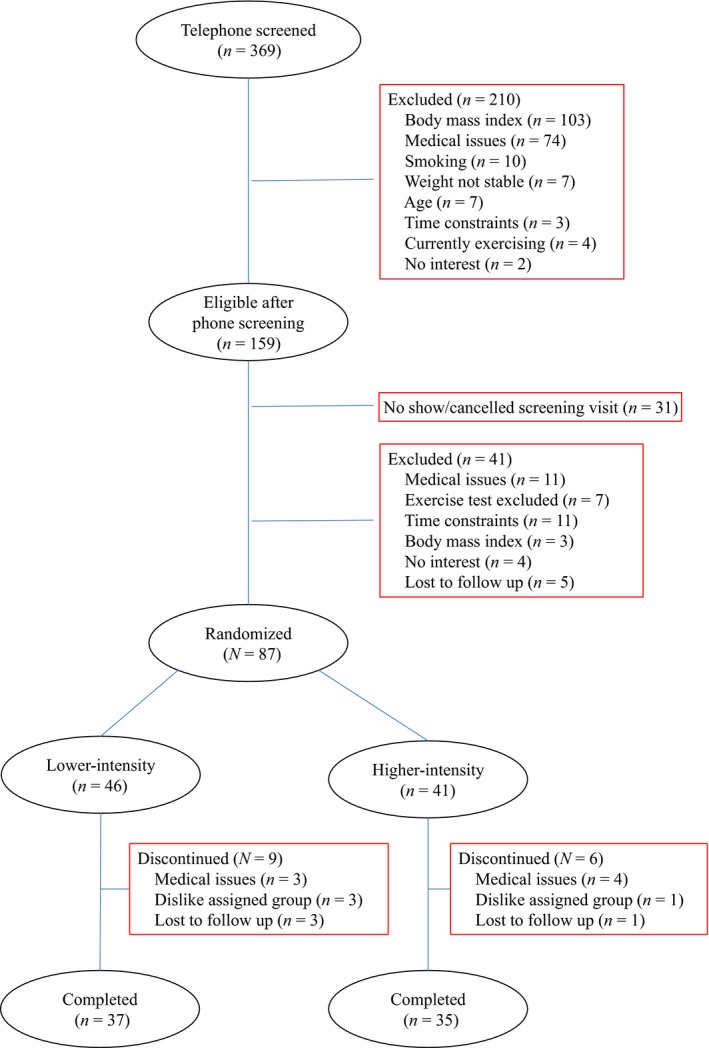
Participant flow diagram.

A total of 369 women were initially screened by telephone (Fig. 1). Of these women, 128 were further screened in our Clinical Exercise Research Center and underwent a medical history review, medical examination, cognitive and depression screening, and fasting blood test. Those with signs of uncontrolled hypertension (blood pressure ≥ 160/90 mmHg); diabetes (fasting glucose ≥ 7.0 mmol·L^−1^); liver, renal, hematologic, or thyroid disorders; cognitive dysfunction (Mini‐Mental State Examination < 24); or depression (Center for Epidemiologic Studies Depression Scale > 16) were excluded. Women showing abnormal response to exercise during a maximum graded exercise test (see below) also were excluded. Upon successful completion of these screenings, 89 women were eligible and 87 completed baseline measurements. All women with abnormal test results were referred for medical attention.

### Randomization and interventions

The 87 women who completed the baseline measurements were randomized to one of two moderate‐intensity walking training programs at lower‐ or higher‐doses (33.6 and 58.8 kJ·kg^−1^ body weight weekly, respectively). The weekly dose of 33.6 kJ·kg^−1^ body weight was estimated to meet the minimum aerobic physical activity recommendation for older adults (U.S. Department of Health and Human Services, 2008; Chodzko‐Zajko et al. 2009). The higher‐dose of 58.8 kJ·kg^−1^ was chosen because this dose was near the maximum exercise dose described in previous exercise studies in older adults (Landers‐Ramos et al. 2016).

All exercise interventions were supervised by exercise trainers and conducted in the Clinical Exercise Research Center at the University of South Carolina. The target intensity of the exercise was 50–55% of each woman's heart rate reserve, calculated as [(peak heart rate−resting heart rate] × intensity (50–55%) + resting heart rate], with resting heart rate and peak heart rate obtained before and during the graded exercise test (see below). Exercise ramped up from a weekly EE of 16.8 kJ·kg^−1^ body weight until the assigned exercise intensity and dosage were reached. The lower‐ and higher‐dose groups reached the exercise target within 5 and 8 weeks, respectively. Each exercise session began with a 3‐min warm‐up and ended with a 3‐min cool down. Women wore heart rate monitors (FT1, Polar, Lake Success, NY) throughout each exercise session to monitor heart rate, which was recorded every 5 min. Blood pressure was measured manually before, at mid‐point, and after each exercise session. To monitor adherence to the exercise prescription, the EE of exercise sessions was calculated using the standard American College of Sports Medicine formula: {0.1 × (speed [miles per hour] × 26.8) + 1.8 × (speed [miles per hour] × 26.8) × grade (%) + 3.5} × weight (kg)/1000 × 5 (L/min) × time (min) (American College of Sports Medicine, 2010). When multiple speeds and grades were performed, the total exercise EE was the sum of all phases.

### Tests and outcome measures

EE, body composition, and peak oxygen consumption (VO_2peak_) were measured before randomization and during the last 2 weeks at end‐intervention.

#### Body composition

Women were in scrubs and without shoes or outer garments to have height and weight measured. Whole‐body fat mass, lean mass, and percentage body fat were measured using dual‐energy X‐ray absorptiometry (enCORE, GE Healthcare model 8743, Waukesha, WI).

#### Graded exercise test

A graded exercise test was performed to identify cardiopulmonary limitations to exercise. Up to 10 min of walking on the treadmill prior to the test was allowed for participants to become familiar with the instrumentation. After a brief rest, women walked at a self‐selected speed with elevation of the treadmill increasing by 2% every 2 min throughout the test. Gas analysis was performed by a metabolic measurement system (TrueOne 2400, Parvo Medics, Salt Lake City, UT), and the 12‐lead electrocardiogram was monitored by a standard system (Quinton Q‐Stress^®^; Cardiac Science, Bothell, WA) continuously during the entire test. Blood pressure was measured during each 2‐min stage. A valid peak volume of oxygen consumption (VO_2peak_) was obtained when at least 2 of the following criteria were met: (1) plateau in VO_2_ (a change < 2 mL/kg/min) with increasing work rate, (2) highest heart rate > 90% of age‐predicted maximum heart rate (220‐age), (3) respiratory exchange ratio ≥ 1.10, and (4) a rate of perceived exertion ≥ 17 on the Borg exertion scale. Using these criteria might underestimate maximum VO_2_ in this population (Poole and Jones 2017); however, the test allowed for the identification and exclusion of women for whom the exercise intervention might not be safe, and also the identification of an intensity range for the exercise intervention.

#### RMR

RMR was measured via indirect calorimetry using a ventilated hood after an overnight fast. Participants rested for approximately 15 min after arriving at the laboratory. Women remained awake, motionless, and in a supine position in a dimly lit and quiet room. During the 30‐min data collection period, the expired air was collected through a one‐way valve and analyzed using a metabolic cart (TrueOne 2400, ParvoMedics, Salt Lake City, UT), which was calibrated on each day of measurement following the manufacturer's instructions. The first and last 5 min of data were excluded to avoid unstable measurements. RMR was calculated from each determination of VO_2_ and VCO_2_ using Weir's (1949) equation. All RMR measurements took place in the morning between 600 and 800 h and at least 24 h after the last bout of any structured exercise.

#### TDEE

TDEE was measured over a 14‐day period using the doubly labeled water method. On day 1, a urine specimen was collected prior to an oral dose of premixed ^18^O and ^2^H labeled water in a body weight adjusted dose. The dosing cup was rinsed twice with drinking water and all water was consumed following consumption of the dose water. On the morning of day 2, the second and third urine samples were collected one hour apart. On the morning of day 15, two more urine samples were collected to close the measurement period. The exact time of each urine sample collection was recorded. Samples were stored at −80°C and analyzed in batches. The enrichment of ^18^O and ^2^H was analyzed using the isotope ratio mass spectrometry, and TDEE was calculated following standard procedures established at the Pennington Biomedical Research Center.

#### Physical activity EE and NEAT

Physical activity EE was calculated as: (TDEE*0.9−RMR), assuming thermic effect of food accounted for 10% of TDEE (Stob et al. 2007; Reed and Hill 1996). NEAT was calculated as physical activity EE minus exercise EE. To calculate the exercise EE during the 14‐day period, a regression equation was developed using VO_2_ and heart rate obtained during each woman's graded exercise test at end‐intervention. The *R* ^2^ value for the regression equations was 91.1 ± 0.1% (mean ± SD). The heart rates during exercise sessions at end‐intervention during the 14‐day period were used in conjunction with each woman's VO_2_ and heart rate regression to calculate the VO_2_, then multiplied by 5 (kcal·L^−1^ O_2_) (Weir 1949) to calculate exercise EE (kcal). Because the women were inactive and their exercise EE was considered 0 prior to the start of the exercise programs, their physical activity EE at baseline equaled NEAT.

#### Accelerometer

Women wore the ActiGraph accelerometer (GT3X+, ActiGraph LLC, Pensacola, FL) on their non‐dominant wrist during the same 14‐day period. They were instructed to keep it on for the entire duration. The GT3X+ is a tri‐axial accelerometer. Mean daily activity counts per minute were calculated using the software provided by the manufacturer (ActiLife 6.9.5, ActiGraph, LLC). Since the activity counts were formed by combining the magnitude of sampled acceleration from all three axes, it was used as a measure of total physical activity. Additionally, using each woman's exercise session times, accelerometer counts during exercise sessions and outside of exercise sessions (nonexercise physical activity counts) were calculated. The nonexercise physical activity counts at baseline were considered equal to the total physical activity counts.

### Statistical analysis

Using information from previous studies (Goran and Poehlman 1992; Meijer et al. 1999), we estimated a difference of 25–30% of baseline NEAT value between the lower‐ and higher‐dose groups in the changes in NEAT from baseline to end‐intervention. Using a NEAT value of 3024 ± 1214 kJ·day^−1^ reported for older adults (Harris et al. 2007), 36 women per group completing the study would provide at least 85% power with an *α* of 0.05 to detect the estimated difference between groups. Variables were reported as means (±SDs) for normally distributed variables, medians (quartiles) for non‐normally distributed variables, or frequencies in percentages. Change values in body weight, TDEE components, and physical activity were calculated using values at end‐intervention minus at baseline. Pearson's correlations were calculated between physical activity and TDEE components at baseline, as well as between changes in physical activity and changes in TDEE components.

Analysis of variance (ANOVA) with repeated measures including an interaction term (group × time) was used to determine if the change over time in TDEE components and physical activity was different between groups. Because body weight and body composition are known to be associated with some TDEE components, body weight changes, or fat mass and fat‐free mass changes were adjusted in the model. To fully account for the role of physical activity in EE responses, baseline physical activity was adjusted as a covariate. Following a significant interaction of baseline physical activity counts × time, data were further analyzed based on tertiles of baseline physical activity counts. One‐way ANOVA was used to determine differences among tertiles, and Tukey's test was used to follow up a significant ANOVA.

Kruskal–Wallis test was used to compare exercise EE and accelerometer counts during exercise sessions between the two groups, because of non‐normal distribution of data. The accelerometer data were missing for three women at baseline and four women at end‐intervention due to accelerometer malfunction. As a result, 66 women had both baseline and end‐intervention data and were included in the analyses that examined interventional effects on physical activity. The analysis based on baseline physical activity tertiles included the 69 women who had baseline data. The sample sizes used are presented in the tables and figure. An *α* level of 0.05 was used to denote statistical significance, and analyses were performed using the SAS software, version 9.4 (SAS Institute, Cary, NC).

## Results

### Participant characteristics

Participant characteristics at baseline are included in Table 1 by assignment group (*n* = 87). These women were older (age = 65.5 ± 4.3 years, mean ± SD), nonobese, and mainly white (83.9%). Their low VO_2peak_ (20.1 ± 3.8 mL·kg^−1^·min^−1^) was reflective of being inactive. There were no differences between the two intervention groups in age, race and ethnicity distribution, body composition, blood pressure, VO_2peak_ or peak heart rate at baseline (*P* > 0.05 for all). The 72 women who have completed the study also had no differences between the two groups in these characteristics (data not shown). The 15 women who dropped out of the study did not differ from those who completed the study at baseline (*P* > 0.05 for all characteristics). Among these 15 women, seven dropped out due to medical issues that were unlikely to be related to the study intervention (three in the lower‐dose and four in the higher‐dose group), four did not like their assigned group (three in the lower‐dose and one in the higher‐dose group), and four were lost to follow up (three in the lower‐dose and one in the higher‐dose group).

**Table 1:** Participant characteristics at baseline by group

	Lower‐dose (*n* = 46)	Higher‐dose (*n* = 41)
Age, years	65.7 ± 4.5	65.2 ± 4.1
Race‐ethnicity, *n* (%)		
Non‐hispanic white	41 (89.1%)	32 (78.0%)
African‐American	4 (8.7%)	6 (14.6%)
Other	1 (2.2%)	3 (4.9%)
Body composition		
Body weight, kg	67.2 ± 9.9	68.2 ± 9.4
BMI, kg·m^−2^	25.5 ± 3.9	25.8 ± 3.0
Fat free mass, kg	41.3 ± 3.9	41.6 ± 5.3
Total fat mass, kg	26.5 ± 7.3	27.3 ± 6.1
Body fat, %	38.4 ± 6.1	39.3 ± 5.6
Systolic blood pressure, mmHg	126 ± 14	127 ± 9
Diastolic blood pressure, mmHg	75 ± 8	75 ± 6
Exercise variables		
VO_2peak_, mL·kg^−1^·min^−1^	20.1 ± 4.1	20.1 ± 3.5
Peak heart rate, beats·min^−1^	159 ± 15	160 ± 12

Values are mean ± SD or *n* (%).

### Exercise intervention effects

Data on exercise prescription and intervention adherence for women who completed the study are shown in Table 2. Mean adherence (expressed as actual/prescribed ratio) to the prescribed exercise intensity was 96.9% and 97.6%, and mean adherence to prescribed exercise dose was 104.4% and 98.7% for the lower‐ and higher‐dose groups, respectively. Women in the lower‐ and higher‐dose groups walked an average of 105 min and 160 min per week, respectively. Most women walked three times a week, although a few women in the higher‐dose group walked four times a week.

**Table 2:** Exercise prescription and intervention adherence for women who completed the study

	Lower‐dose (*n* = 37)	Higher‐dose (*n* = 35)
Exercise intensity		
Prescribed heart rate, beats·min^−1^	118 ± 11	119 ± 7
Actual heart rate at mid‐exercise session, beats·min^−1^	114 ± 11	116 ± 10
Adherence to intensity (actual/prescribed heart rate), %	96.9 ± 7.6	97.6 ± 8.4
Exercise dose		
Prescribed weekly exercise dose, kJ	2268 ± 328	3948 ± 516
Actual weekly exercise dose,^a^ kJ	2365 ± 386	3902 ± 643
Adherence to exercise dose (actual/prescribed dose), %	104.4 ± 8.5	98.7 ± 7.5

Values are mean ± SD.

a Calculated using American College of Sports Medicine formula (American College of Sports Medicine, 2010).

The two exercise groups did not differ in the changes in body composition variables (*P* for group × time interaction > 0.05 for all). In the total sample, there were small but statistically significant reductions in body weight (Δ = −0.8 ± 2.1 kg, *P* = 0.002), BMI (Δ = −0.4 ± 0.8 kg·m^−2^, *P* < 0.0001), fat mass (Δ = −0.9 ± 1.8 kg, *P* < 0.0001), and percent body fat (Δ = −0.7 ± 1.6%, *P* = 0.0002). Fat‐free mass remained unchanged (*P* = 0.21). Change in VO_2peak_ differed significantly between the two groups (*P* = 0.017 for group × time interaction); increases occurred in both groups but with different magnitude (lower‐dose: Δ = 0.6 ± 2.8 mL·kg^−1^·min^−1^, *P* < 0.001; higher‐dose: Δ = 2.3 ± 3.1 mL·kg^−1^·min^−1^, *P* < 0.001).

Components of TDEE and physical activity at baseline and end‐intervention by group are shown in Table 3. At baseline, the two groups had similar TDEE and RMR, and these EE measures did not change with exercise training (*P* > 0.05 for both). Physical activity EE, NEAT, and physical activity counts obtained from accelerometers (as a measure of total physical activity), were similar for the two groups at baseline (*P* > 0.05 for all). Despite the difference in exercise dose at end‐intervention during the 14‐day doubly labeled water measurement period (*P* = 0.005 for Kruskal–Wallis test), there were no differences in changes in physical activity EE, NEAT, or physical activity counts (total or nonexercise) in the two groups from baseline to end‐intervention (*P* for group × time interaction > 0.05 for all). In the two groups combined, physical activity EE increased significantly (*P* = 0.034). However, total or nonexercise physical activity counts did not change with exercise training (*P* = 0.21 and 0.40, respectively), and NEAT did not change (baseline NEAT was assumed to equal to physical activity EE) (*P* = 0.93). At end‐intervention, neither accelerometer counts during exercise sessions (*P* = 0.96 for Kruskal–Wallis test) nor nonexercise physical activity counts (*P* = 0.57) differed between the two groups.

**Table 3:** Energy expenditure and physical activity at baseline and end‐intervention by group

	Lower‐dose (*n* = 37)	Higher‐dose (*n* = 35)
TDEE, kJ·day^−1^		
Baseline	8765 ± 949	8610 ± 1285
End‐intervention	8828 ± 1226	8900 ± 1604
RMR, kJ·day^−1^		
Baseline	5200 ± 664	5069 ± 685
End‐intervention	5032 ± 617	5040 ± 769 322 (204, 430)^c^
Exercise EE,^a^ end‐intervention, kJ·day^−1^	189 ± 89	
Physical activity EE, kJ·day^−1^		
Baseline	2668 ± 808	2680 ± 865
End‐intervention	2871 ± 952	2969 ± 991
NEAT, end‐intervention, kJ·day^−1^	2680 ± 922	2644 ± 963
Total physical activity,^b^ counts per minute per day		
Baseline	1534 ± 417	1634 ± 442
End‐intervention	1599 ± 392	1656 ± 437
Nonexercise physical activity, end‐intervention,^b^ counts per minute per day	1590 ± 391	1637 ± 435
Exercise, end‐intervention,^b^ accelerometer counts per minute per day	2114 (1470, 4197)	2423 (1741, 3609)

Data are presented as mean ± SD, or median (lower and upper quartiles). TDEE, total daily energy expenditure; RMR, resting metabolic rate; NEAT, nonexercise activity thermogenesis.

a Calculated using VO_2_ and heart rate regression developed for each woman using data from graded exercise test at end‐intervention.

b *n* = 33 for lower‐dose group and *n* = 33 for higher‐dose group.

c *P* = 0.005 for Kruskal–Wallis test for comparison between groups.

After adjustment for body weight changes, physical activity EE was no longer greater than baseline; TDEE, NEAT, and physical activity counts (total or nonexercise) remained unchanged after the intervention (*P* > 0.05 for all). However, a significant weight change × time interaction was found for RMR (*P* = 0.0098). This was supported by a significant association between changes in RMR and changes in body weight (*r* = 0.30, *P* = 0.01). After adjustment for fat‐free mass and fat mass changes instead of body weight changes, TDEE, RMR, NEAT, and physical activity counts were unchanged (*P* > 0.05 for all), but physical activity EE was still greater than baseline (*P* = 0.045).

### Effects of baseline physical activity

In order to better describe the role of physical activity, we also examined the impact of baseline physical activity (using physical activity counts obtained from accelerometers as a measure of total physical activity) on components of TDEE. At baseline, there were significant associations between physical activity counts and TDEE (*r* = 0.55, *P* < 0.0001) and physical activity counts and physical activity EE (*r* = 0.66, *P* < 0.0001), but not between physical activity counts and RMR (*r* = 0.073, *P* = 0.55). The changes in physical activity counts, however, were not associated with changes in TDEE, RMR, physical activity EE, or NEAT (*P* > 0.10 for all).

After adjustment for baseline physical activity counts in the ANOVA models with repeated measures examining components of TDEE after exercise training, TDEE was still unchanged (*P* = 0.13). However, a significant baseline physical activity counts × time interaction was found for RMR, physical activity EE, NEAT, and total and nonexercise physical activity counts (*P* < 0.05 for all), suggesting that the changes in these variables after exercise training differed depending on baseline physical activity levels. These significant interactions still existed after further adjustment for body weight change or fat mass and fat‐free mass changes (*P* < 0.05 for all).

**Figure 2:**
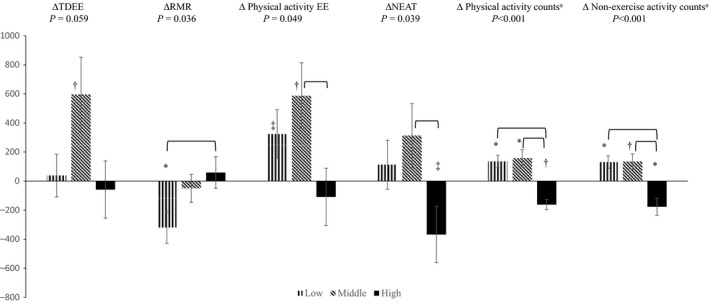
Changes in energy expenditure (kJ·day^−1^) and physical activity (counts·min^−1^·day^−1^) by tertiles of baseline physical activity counts (*n* = 23 in each tertile). Change values (Δ) are calculated as values at end‐intervention minus at baseline. TDEE, total daily energy expenditure. RMR, resting metabolic rate. NEAT, nonexercise activity thermogenesis. Values are mean ± standard error. *P* values are for comparisons among tertiles. Horizontal parenthesis indicates significant difference between the two respective tertiles. ^a^, *n* = 21, 23, and 22 in low, middle, and high tertile, respectively. *, *P* < 0.01 for within tertile change after exercise intervention. ^†^, 0.01 < *P *<* *0.05 for within tertile change after exercise intervention. ^‡^, 0.05 < *P *<* *0.10 for within tertile change after exercise intervention.

Therefore, data were categorized into tertiles based on baseline physical activity counts (mean daily physical activity <1312, 1312–1731, and >1731 counts per minutes per day for the three tertiles, respectively). Among the tertiles, there were significant differences in the changes from baseline to end‐intervention in RMR, physical activity EE, NEAT, and total and nonexercise physical activity counts (*P* < 0.05 for all), but no differences in TDEE (*P* = 0.059) (Fig. 2). Specifically, RMR changed differently in the low compared to the high tertile (*P* = 0.033). Both physical activity EE and NEAT changed differently in the middle compared to the high tertile (*P* = 0.040 and 0.042, respectively). Total and nonexercise physical activity counts changed differently in the low and middle compared to the high tertile (*P* ≤ 0.001 for all). Within the low tertile, RMR decreased and total and nonexercise physical activity counts increased (*P* < 0.01 for all). Within the middle tertile, TDEE, physical activity EE, and total and nonexercise physical activity counts increased (*P* < 0.05 for all). Within the high tertile, total and nonexercise physical activity counts decreased (*P* < 0.05 for both).

Further adjustment for body weight changes did not affect the comparisons among tertiles. Most within‐tertile changes were not affected, except that RMR no longer decreased in the low tertile. Adjustment for changes in fat free mass and fat mass instead of body weight changes resulted in similar findings for comparisons among tertiles, follow‐up pairwise comparisons, and within‐tertile changes. The only difference was that in the middle tertile, nonexercise physical activity was no longer statistically changed (*P* = 0.065) after adjustment for fat free mass and fat mass changes.

## Discussion

This is one of the first studies using solid measurement methods of EE (including the gold standard doubly labeled water method for assessing free‐living TDEE) and simultaneously accessing physical activity using a randomized design in older women to investigate EE and physical activity responses to aerobic exercise training. We found that TDEE, RMR, NEAT, and total and nonexercise physical activity (counts by accelerometers) did not change. Also, participation in aerobic exercise interventions of two different doses did not result in different changes in any of the TDEE components or physical activity. The intriguing finding was that the changes in several of these variables, except TDEE, appeared to differ depending on the amount of baseline physical activity.

Our results of no difference in changes in TDEE and its components between the two groups of different exercise doses were similar to a few previous studies which also investigated the effects of various doses of exercise training on NEAT or nonexercise physical activity. Specifically, no differences in nonexercise physical activity has been shown after exercise training at 16.8, 33.6, and 50.4 kJ·kg^−1^ body weight weekly (Church et al. 2009), 3780 and 7560 kJ weekly (Rosenkilde et al. 2012), or 5023 and 8372 kJ weekly (Hollowell et al. 2009). Participants in our study were older than in these studies; therefore, extending the findings to an older population.

Several previous studies involving older adults suggested decreased nonexercise physical activity (Meijer et al. 1999, 2000) and/or NEAT (Goran and Poehlman 1992; Morio et al. 1998; Colley et al. 2010) following exercise intervention, but others reported increased or unchanged nonexercise physical activity (Washburn and Ficker 1999; Fujita et al. 2003; Church et al. 2009). Our study showed that NEAT and nonexercise physical activity did not change in the overall sample. However, nonexercise physical activity counts decreased in women whose physical activity at baseline was within the high tertile, but increased in those whose physical activity was in the low or middle tertile. NEAT showed a similar trend, although the change in NEAT in each of the tertiles did not reach statistical significance. In fact, previous studies have reported different changes in nonexercise physical activity in some subsets of data. For example, 44% of postmenopausal women experienced a decrease in spontaneous physical activity (similar to nonexercise physical activity), but the other 56% experienced an increase (Di Blasio et al. 2012). Another study found increased nonexercise physical activity in a small subset (*n* = 8) but no change in the entire sample (*n* = 32) (Meijer et al. 1991). None of the previous exercise intervention studies examined whether baseline physical activity affected the changes in nonexercise physical activity or NEAT. Therefore, our data do not conflict with previous studies, but may have provided a reason for the inconsistent results by demonstrating baseline physical activity modulated the responses of physical activity and NEAT to aerobic exercise training.

Interestingly, a pattern in EE and physical activity changes across the tertiles of baseline physical activity was shown in this study. Significantly different changes in TDEE components and physical activity were found among the tertiles, which resulted in an increased TDEE in the middle tertile only. The unchanged TDEE in the low and high tertiles appeared to be due to different changes in TDEE components, primarily a reduced RMR in the low tertile and a reduced NEAT in the high tertile (although it did not reach statistical significance). Recently, a constrained TDEE model has been proposed (Pontzer 2015; Pontzer et al. 2016). This model suggests that TDEE is maintained within a narrow range, and that it increases with physical activity at lower activity levels but plateaus at higher activity levels. In cross‐sectional data collected from populations ranging from low to high daily physical activity levels, body size adjusted‐TDEE plateaued over the upper range of daily physical activity, and 230 counts per minutes per day (by Actical, Philips Respironics) was described as the change point of the physical activity and TDEE association (Pontzer et al. 2016). Due to different models of accelerometers, it is difficult to directly compare the actual count values in our study to that study. However, our finding that TDEE increased in women whose baseline physical activity was in the middle tertile but did not change in the high tertile supports the constrained TDEE model.

Conversely, TDEE did not increase significantly in the low baseline physical activity tertile, possibly due to reduction in body weight, which resulted in significantly reduced RMR. This notion is supported by the findings that no differences were found between the low and middle tertiles in changes in total physical activity or physical activity EE, and that RMR was no longer reduced after adjustment for body weight changes. Further examination revealed a trend toward a greater weight loss in this subgroup of women (−1.2 ± 1.4 kg) compared to the middle and high tertiles (−0.5 ± 2.7 and −0.8 ± 1.9 kg, respectively). These findings are important, suggesting that in the most sedentary individuals in the population, exercise training could lead to greater weight loss than in more active individuals.

Simultaneously examining several components of TDEE and measuring physical activity using accelerometers allowed us to examine both the physiological and the behavioral aspects of the body's responses to aerobic training. The physical activity counts include any physical activity that created an acceleration above the data collection threshold of the accelerometers; thus, it could be considered a measure of total physical activity, that is, behavioral physical activity participation. This behavioral aspect of physical activity increased with both lower‐ and higher‐dose exercise training in women in the low and middle tertiles, but decreased in the high tertile of baseline physical activity. Also, the results of nonexercise and total physical activity counts were very similar, which strengthened this finding. In contrast, physical activity EE and NEAT are influenced not only by the amount of physical activity (i.e., the behavioral aspect), but also by body mass and the energy cost associated with moving per unit of body mass (i.e., physiological aspect) in specific movements. Additional energy cost, such as energy cost of posture maintenance, static exercise, and excess postexercise oxygen consumption (EPOC), may also be included in the calculated physical activity EE and NEAT. The differences in these measures likely contribute to the inconsistent findings of previous studies that examined only nonexercise physical activity or NEAT. As we have shown, physical activity measured by accelerometers and calculated EE of physical activity or NEAT do not always change in the same fashion.

A few points should be noted when interpreting the results of this study. Accelerometers have their limitations, and they are not able to detect muscle activities toward postural efforts against gravity, fidgeting, static exercise, or physical activity below their detection threshold. The accelerometer was worn on the wrist in order to increase wear time; however, this might result in inaccurate measurement of physical activity. For example, some women held onto the treadmill periodically during the exercise sessions, which may lead to underestimated counts on a wrist‐worn accelerometer. Therefore, results from this study cannot be compared directly with other studies in which accelerometers were worn on the hip. However, these limitations likely have little impact on our conclusion, because activities that are not captured by accelerometry contribute to the physiological aspect of physical activity (calculated physical activity EE and NEAT). The doubly labeled water method, although considered the gold standard for measuring TDEE, has a small 5–10% measurement error (Schoeller 1999; Sagayama et al. 2016). The error rates of the calculated TDEE components, physical activity EE and NEAT, likely would be greater than the measured variables (TDEE and RMR) from which they were derived. Also, we assumed the exercise EE and accelerometer counts were 0 at baseline. Given the exclusion criterion of less than 20 min of structured exercise for 3 times/week, it is possible that exercise EE and accelerometer counts were greater than 0 for some women at baseline. Nonetheless, this may only slightly influence the magnitude of NEAT change because very few women reported participating in structured exercise before the study. Additionally, because our participants were nonobese older women, caution should be made in extrapolating our results to obese individuals or other age groups. We did not have a no‐exercise control group, which limited our ability to determine whether other factors might have contributed to the results. The analysis based on baseline physical activity was not a priori and the sample size estimation was not based on this analysis; therefore it should be considered as exploratory in nature. Finally, there were large individual variances in EE, and our conclusions were drawn from data at the sample mean level. The factors that determine the individual responses need further investigation.

In summary, our study found that exercise training did not change TDEE, RMR, NEAT, total physical activity or non‐exercise physical activity in the total sample, and the two doses of exercise did not impact these variables differently. Our exploratory analysis suggested different responses in TDEE components in women whose baseline physical activity was in the high, middle, and low tertiles. Taken together, these results show that EE and physical activity responses to exercise participation are not uniform; baseline characteristics of participants may be important factors influencing an individual's response to exercise, and should be considered to help personalize treatment. Future studies are needed to test whether our findings remain true, especially in obese individuals, given the common practice to include an exercise intervention in strategies to induce weight loss. Future studies are also needed to identify other factors that may also influence the EE and physical activity responses to exercise.

## Conflict of Interest

None.
